# Supplementary material for: Genomic and phenotypic stability of fusion-driven pediatric sarcoma cell lines
Source: Nat Commun. 2025 Jan 3;16:380. doi: 10.1038/s41467-024-55340-5 (PMC11699042; doi:10.1038/s41467-024-55340-5)
Supplement: Supplementary file 1 — Supplementary Information [file 41467_2024_55340_MOESM1_ESM.pdf]

SUPPLEMENTARY FIGURES

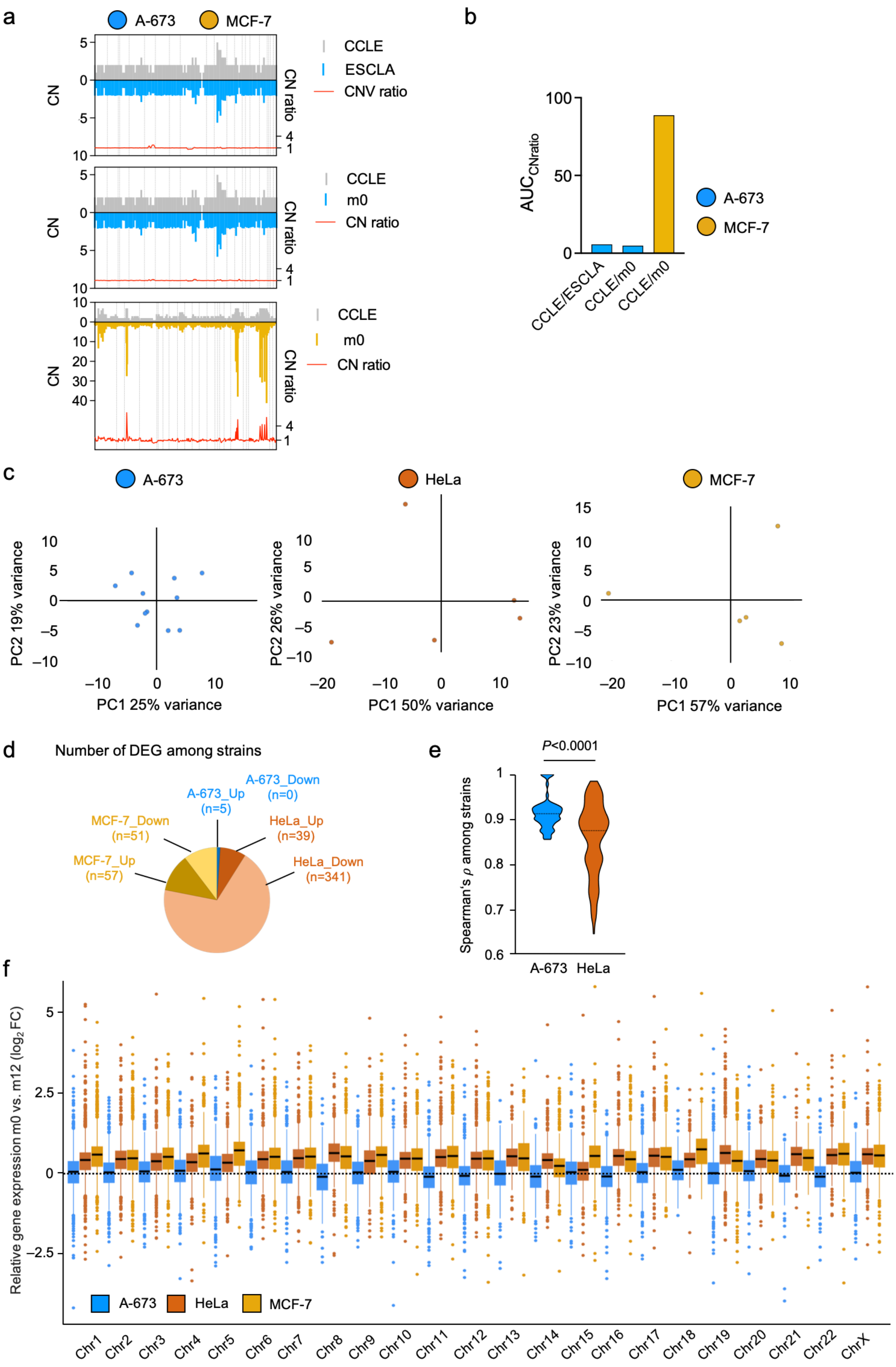

Supp. Fig. 1 | Fusion-driven pediatric sarcoma cell line strains display stable and homogenous genomes and transcriptomes. a. Mirror plot shows total copy number (CN) (y-axis, left) and CN ratio (y-axis, right) across the genome of A-673 or MCF-7 cell strains. The grey bars represent the total CN derived from the Cancer Cell Line Encyclopedia (CCLE) (A-673 and MCF-7 strains). The blue bars represent the total CN estimated from Whole Genome Sequencing (WGS) data processed using the ACEseq algorithm from BioProject PRJNA610192 (ESCLA), A-673_m0, or MCF-7_m0. The red line denotes the CN ratio ($WGS_{CN}/CCLE_{CN}$). The x-axis represents chromosomal positions across the genome. Vertical gray dotted lines indicate chromosome limits. **b**. Plot shows area under the curve for each calculated CN ratio in a. for A-673 (blue) or MCF-7 (yellow) **c**. Independent PCA of 11 A-673, five HeLa, and five MCF-7 strains. Axes depict the variance percentages across each cell line's strains. **d.** Pie chart depicts the number of up- and down-regulated DEG emerging from each comparison. **e.** Gene-specific Spearman's ρ across A-673 and HeLa cell line strains from the combined transcriptomic datasets in Fig 1f. Dotted black lines show the median (one-sided Wilcoxon rank-sum test). **f.** Relative gene expression of A-673, HeLa, and MCF-7 cell lines after long-term culture for twelve months (m12 vs m0) per chromosome. Boxplots display the interquartile range and the mean.

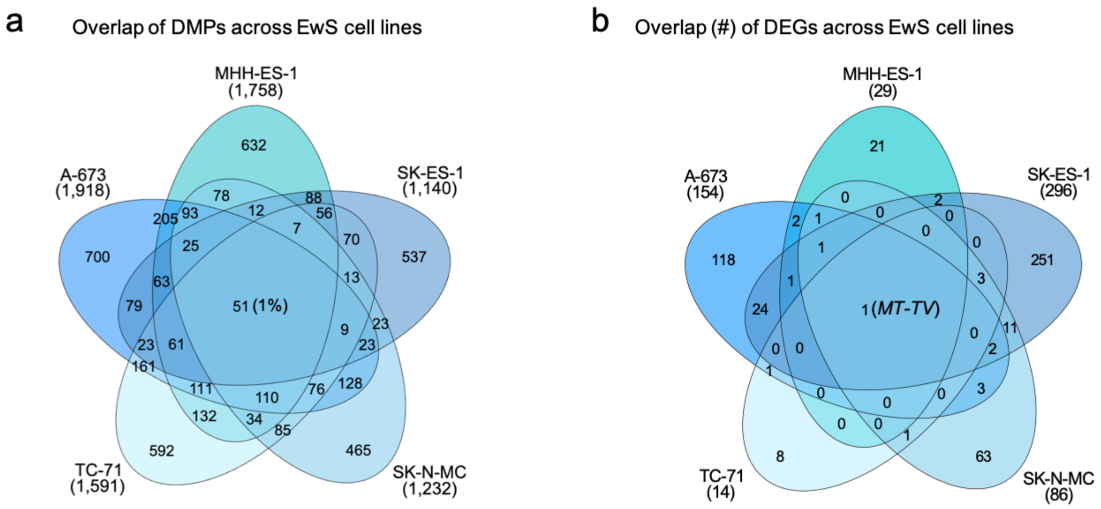

Supp. Fig. 2 | In-depth analysis of stability on individual EwS cell lines. a. Venn diagram displaying the overlap in differentially methylated CpG sites across all EwS cell lines after 12 months in continuous culture (m12/m0). **b**. Venn diagram displaying the overlap in differentially expressed genes across all EwS cell lines after 12 months in continuous culture (m12/m0).
