# Supplementary material for: Genomic and phenotypic stability of fusion-driven pediatric sarcoma cell lines
Source: Nat Commun. 2025 Jan 3;16:380. doi: 10.1038/s41467-024-55340-5 (PMC11699042; doi:10.1038/s41467-024-55340-5)
Supplement: Supplementary file 2 — Description of Additional Supplementary Files [file 41467_2024_55340_MOESM2_ESM.pdf]

Description of Additional Supplementary Files

Supplementary Data 1 | Description of compounds included in the drug screening

Supplementary Data 2 | ns-SNPs displaying a varying SNP status in EwS cell lines

Supplementary Data 3 | DMPR in EwS cell lines cultured over 12 months.

Supplementary Data 4 | DEG in EwS cell lines cultured over 12 months
