# Supplementary material for: Genomic and phenotypic stability of fusion-driven pediatric sarcoma cell lines
Source: Nat Commun. 2025 Jan 3;16:380. doi: 10.1038/s41467-024-55340-5 (PMC11699042; doi:10.1038/s41467-024-55340-5)
Supplement: Supplementary file 7 — Reporting Summary [file 41467_2024_55340_MOESM7_ESM.pdf]

nature portfolio

Corresponding author(s): Florencia Cidre-Aranaz

Last updated by author(s): Oct 25, 2024

Reporting Summary

Nature Portfolio wishes to improve the reproducibility of the work that we publish. This form provides structure for consistency and transparency in reporting. For further information on Nature Portfolio policies, see our Editorial Policies and the Editorial Policy Checklist.

Statistics

For all statistical analyses, confirm that the following items are present in the figure legend, table legend, main text, or Methods section.

n/a	Confirmed	
☐	☒	The exact sample size (*n*) for each experimental group/condition, given as a discrete number and unit of measurement
☐	☒	A statement on whether measurements were taken from distinct samples or whether the same sample was measured repeatedly
☐	☒	The statistical test(s) used AND whether they are one- or two-sided<br>*Only common tests should be described solely by name; describe more complex techniques in the Methods section.*
☐	☒	A description of all covariates tested
☐	☒	A description of any assumptions or corrections, such as tests of normality and adjustment for multiple comparisons
☐	☒	A full description of the statistical parameters including central tendency (e.g. means) or other basic estimates (e.g. regression coefficient) AND variation (e.g. standard deviation) or associated estimates of uncertainty (e.g. confidence intervals)
☐	☒	For null hypothesis testing, the test statistic (e.g. *F*, *t*, *r*) with confidence intervals, effect sizes, degrees of freedom and *P* value noted<br>*Give P values as exact values whenever suitable.*
☒	☐	For Bayesian analysis, information on the choice of priors and Markov chain Monte Carlo settings
☒	☐	For hierarchical and complex designs, identification of the appropriate level for tests and full reporting of outcomes
☐	☒	Estimates of effect sizes (e.g. Cohen's *d*, Pearson's *r*), indicating how they were calculated

Our web collection on statistics for biologists contains articles on many of the points above.

Software and code

Policy information about availability of computer code

Data collection	Illumina Infinium GSA v3.0 gene annotation file (GSE111485_rpkms) was retrieved from Gene Expression Omnibus.
Data analysis	Data analysis and visualization was performed primarily in R v.3 and v.4 with R packages (specified in detail in the methods section of the manuscript). Additional data visualization was performed using PRISM 9 (GraphPad Software Inc., CA, USA). Venn diagrams were plotted using Affinity Designer 2, version 2.4.2.

For manuscripts utilizing custom algorithms or software that are central to the research but not yet described in published literature, software must be made available to editors and reviewers. We strongly encourage code deposition in a community repository (e.g. GitHub). See the Nature Portfolio guidelines for submitting code & software for further information.

Data

Policy information about availability of data

All manuscripts must include a data availability statement. This statement should provide the following information, where applicable:

- Accession codes, unique identifiers, or web links for publicly available datasets
- A description of any restrictions on data availability
- For clinical datasets or third party data, please ensure that the statement adheres to our policy

Original data that support the findings of this study was deposited at the National Center for Biotechnology Information (NCBI) GEO under accession numbers GSE270195, GSE268437, GSE264509, and under BioProject PRJNA1160032. Source data are provided with this paper.

Research involving human participants, their data, or biological material

Policy information about studies with human participants or human data. See also policy information about sex, gender (identity/presentation), and sexual orientation and race, ethnicity and racism.

Reporting on sex and gender	Not applicable
Reporting on race, ethnicity, or other socially relevant groupings	Not applicable
Population characteristics	Not applicable
Recruitment	Not applicable
Ethics oversight	not applicable

Note that full information on the approval of the study protocol must also be provided in the manuscript.

Field-specific reporting

Please select the one below that is the best fit for your research. If you are not sure, read the appropriate sections before making your selection.

☒ Life sciences ☐ Behavioural & social sciences ☐ Ecological, evolutionary & environmental sciences

For a reference copy of the document with all sections, see nature.com/documents/nr-reporting-summary-flat.pdf

Life sciences study design

All studies must disclose on these points even when the disclosure is negative.

Sample size	In vitro sample sizes were based on previous similar studies that have given statistical results.
Data exclusions	Data exclusions were solely performed during quality control measurements in (epi)genomic, transcriptomic, and phenotypic analyses. This step was taken to ensure statistical integrity (such as removal of missing data and minimally expressed genes)
Replication	Drug screening analysis featured four biological replicates per screening. RNA samples from three biological replicates were used in two technical replicates. DNA was analyzed in two and a single technical replicates for Illumina EPIC Array and GSA,respectively.
Randomization	not applicable
Blinding	No blinding was performed

Reporting for specific materials, systems and methods

We require information from authors about some types of materials, experimental systems and methods used in many studies. Here, indicate whether each material, system or method listed is relevant to your study. If you are not sure if a list item applies to your research, read the appropriate section before selecting a response.

Materials & experimental systems

n/a	Involved in the study	
☒	☐	Antibodies
☐	☒	Eukaryotic cell lines
☒	☐	Palaeontology and archaeology
☒	☐	Animals and other organisms
☒	☐	Clinical data
☒	☐	Dual use research of concern
☒	☐	Plants

Methods

n/a	Involved in the study	
☒	☐	ChIP-seq
☒	☐	Flow cytometry
☒	☐	MRI-based neuroimaging

Eukaryotic cell lines

Policy information about cell lines and Sex and Gender in Research

Cell line source(s)	MCF-7, MHH-ES-1, SK-ES-1, SK-N-MC, and TC-71 cell lines were purchased from the German Collection of Microorganism and Cell Cultures (DSMZ). A-673 was purchased from ATCC.
Authentication	All cell lines were authenticated using STR-profiling.
Mycoplasma contamination	All cell lines tested negative for mycoplasma contamination.
Commonly misidentified lines (See ICLAC register)	SK-N-MC: The SK-N-MC cell line is listed in the database of commonly misidentified cell lines, ICLAC (http://iclac.org/databases/cross-contaminations), as it was initially described to be a neuroblastoma cell line. Indeed, it is a Ewing sarcoma cell line expressing the pathognomonic fusion oncogene EWSR1-FLI1.

Plants

Seed stocks	not applicable
Novel plant genotypes	not applicable
Authentication	not applicable
